# Carambolaside W Inhibited H1N1 Influenza Virus-Induced Oxidative Stress through STAT-3/BCL-XL Signaling Pathway

**DOI:** 10.3390/v15091858

**Published:** 2023-08-31

**Authors:** Jingyao Su, Jia Lai, Jiali Li, Xia Liu, Haitian Chen, Chuqing Li, Bing Zhu, Xuchao Jia, Yinghua Li

**Affiliations:** 1 Center Laboratory, Guangzhou Women and Children’s Medical Center, Guangzhou Medical University, No. 318 Renminzhong Road, Yuexiu District, Guangzhou 510120, China; sujingyao@stu.gzhmu.edu.cn (J.S.); 2022210293@stu.gzhmu.edu.cn (J.L.);; 2 Sericultural & Agri-Food Research Institute Guangdong Academy of Agricultural Sciences/Key Laboratory of Functional Foods, Ministry of Agriculture and Rural Affairs/Guangdong Key Laboratory of Agricultural Products Processing, Guangzhou 510610, China

**Keywords:** carambolaside W, H1N1 influenza, oxidative stress, apoptosis

## Abstract

The H1N1 influenza virus is highly infectious and pathogenic, and in recent years, it has often presented seasonal mass outbreaks of infection. People infected with H1N1 will develop a high fever and other respiratory infection symptoms. If not treated in time, complications such as pneumonia may occur. In this study, we focused on developing drugs that can effectively fight against with H1N1 virus. A flavonoid glycoside was extracted from the carambola, then characterized by HR-ESI-MS with the molecular formula C_47_H_58_O_2_, and named carambolaside W. The flavonoid glycosides were found to have good anti-H1N1 influenza virus effects. In this study, we verified that carambolaside W has low toxicity and can effectively inhibit influenza virus replication *in vitro*. H1N1 virus infection induces intracellular oxidative stress damage to accelerate disease progression. The results showed that carambolaside W effectively inhibited the oxidative stress caused by H1N1 infection. The Western blot assay also revealed that carambolaside W alters the expression of apoptosis-related proteins *in vitro* and exerts a good anti-H1N1 influenza virus effect. In summary, carambolaside W is a low-toxicity natural flavonoid that can effectively treat the H1N1 influenza virus as a potential anti-H1N1 virus agent.

## 1. Introduction

The H1N1 influenza virus causes acute respiratory infections and has a complex structure consisting of an envelope, a matrix protein, and a protein core. The innermost layer is a ribonucleoprotein complex composed of RNA, Polymerase basic protein (PB1, PB2), Polymerase acidic protein (PA), and Nuclear protein (NP), which forms the core of the virus particle [1,2,3]. Influenza A virus (IAV) is a pathogen that can be classified into different subtypes based on the surface viral glycoproteins Hemagglutinin (HA) and Neuraminidase (NA) [4,5]. IAV causes severe respiratory symptoms in humans and animals; it is highly contagious and can be transmitted through the mouth, nose, air, droplets, etc. [6]. Vaccination is currently the main measure to prevent infection, but the frequent antigenic drift and antigenic translocation of influenza virus surface proteins have led to limitations in the development, application, and promotion of vaccines [7,8,9]. Antiviral drugs currently used in clinical practice include inhibitors of the neuraminidase protein, the integral membrane proton channel M2 protein. In addition, because the structure of RNA polymerase is conserved in different viral strains, RNA-dependent protein RNA polymerase inhibitors, have also been used in a large number of clinical applications, such as the PB1 inhibitor favipiravir, the PB2 inhibitor Pimodivir, and the PA inhibitor Baloxavir Marboxil [10,11]. However, with the long-term use of these drugs in clinical therapy and the high mutation rate of influenza viruses, drug-resistant strains continue to emerge [12]. The development of new anti-influenza viral drugs continues to be a major effort by researchers.

The fruit of *Averrhoa carambola* L. (Oxalidaceae), commonly known as carambola, is a fruit commonly consumed in many countries. In previous studies, it was verified by phytochemistry and pharmacology that the leaves, root extracts, and fruits of carambola are rich in saponins, alkaloids, flavonoids, and tannins [13,14,15]. In addition, carambola is an important source of vitamins and minerals that the body needs, such as potassium, phosphorus, magnesium, and vitamin C, which contribute to the strengthening of the immune system [16,17]. Cabrini et al. showed that the anti-inflammatory activity of carambola extracts contributed to the reduction of inflammatory skin conditions. When the ethanolic extract of the leaves of the carambola plant was applied to the skin, inflammation in the skin of mice was reduced, and eczema symptoms were gradually reduced [18]. Combining the above characteristics, carambola has a strong antioxidant function, can enhance the body’s immune function to a certain extent, and plays a role in regulating the inflammatory response [19,20]. In this study, a new flavonoid glycoside compound was extracted from carambola fruit, named carambolaside W. The study on the treatment *in vitro* of the H1N1 influenza virus hopes to use the antioxidant and anti-inflammatory properties of carambola to inhibit the oxidative stress brought by the H1N1 influenza virus and the cytokine storm caused by immune deficiency [21,22,23] and provide a potential drug that could be effective against the H1N1 influenza virus.

## 2. Materials and Methods

### 2.1. General Experimental Procedures

NMR spectra were obtained on a Bruker Ascend-500 spectrometer (Billerica, MA, USA) using solvent peaks as references. HRESIMS spectra were measured on a Bruker Maxis mass spectrometer (Billerica, MA, USA). HPLC was performed on an LC3000 set connected to a UV3000 scanning spectrophotometer detector (Beijing ChuangXin TongHeng Sci. & Tech. Co., Beijing, China), and the columns used were Cosmosil 5C18-MS-II (Suzhou, China), 5 μm, 250 mm × 4.6 mm i.d. for analysis and 250 mm × 20 mm i.d. for preparation (Nacalai Tesque Inc., Kyoto, Japan). Medium-pressure liquid chromatography (MPLC) was performed on an EZ Purifier (Leisure Science, Suzhou, China), and the column used was Chromatorex RP-18 SMB100, 20–45 µm, 400 mm × 25 mm i.d. (Fuji Silysia Chemical, Aichi, Japan) [24,25]. Silica gel (100–200 mesh, Qingdao Haiyang Chemical Co., Qingdao, China), and Sephadex LH-20 (GE Healthcare Bio-Sciences AB, Uppsala, Sweden) were used for CC.

### 2.2. Materials

Madin-Darby canine kidney cells (MDCK) were purchased from the American Type Culture Collection (ATCC CCL-34TM). Carambolaside W was kindly provided by the Guangdong Academy of Agricultural Sciences. The typed H1N1 influenza virus was provided by the central laboratory of Guangzhou Women’s and Children’s Medical Center, and it was sequenced and determined to be influenza A/Puerto Rico/8/1934 (H1N1) virus (PR8). The influenza viruses used in the experiments in this manuscript were all PR8, Cell Counting Kit-8 (CCK8), mitochondrial membrane potential assay kit (JC-1), Annexin-V-FITC staining kit, and BCA protein assay kit were purchased from Beyotime (Shanghai, China). Cell Signaling Technology (Danvers, MA, USA) provided anti-C-PARP antibody, anti-PARP antibody, anti-STAT3 antibody, anti-Bcl-XL antibody, anti-β-actin, and anti-mouse IgG antibody for Western blotting. The DAPI stain for cell nuclear localization was purchased from SIGMA(Darmstadt, German). Fetal bovine serum (FBS), trypsin, and Dulbecco’s modified Eagle’s medium (DMEM) were purchased from Gibco (Carlsbad, CA, USA). Oxidative data from the BD FACSCanto II flow cytometer (Franklin Lakes, NJ, USA). 

### 2.3. Plant Material

Fresh ripe carambola is purchased from the market in Tianhe District, Guangzhou. The species was botanically authenticated to be *Averrhoa carambola* by Prof. Jianwei Chi in the Sericultural & Agri-Food Research Institute, Guangdong Academy of Agricultural Sciences. A voucher specimen (No. 200016) was deposited at our laboratory.

### 2.4. TCID50 of the Influenza Virus

MDCK cell suspension at a density of 7 × 10^4^ was first added to the 96-well plate and incubated in an incubator at 37 °C with 5% CO_2_. After diluting the virus inoculum to concentrations of 10^−1^, 10^−2^, 10^−3^, 10^−4^, 10^−5^, 10^−5^, 10^−6^, 10^−7^, 10^−8^, 10^−9^, and 10^−10^, when the cells were grown flat to a density of 70%, the assay was started [26]. The virus was adsorbed in 96-well plates for 2 h, and then the virus culture medium was removed. The virus cytopathic effect was observed after 48 h. Finally, cytopathic effects were observed and counted, and the infectious dose (TCID50) was calculated by using the Reed-Muench method to obtain the toxicity of H1N1 [24].

### 2.5. Detection of Drug Toxicity and Drug Therapeutic Capacity

Firstly, MDCK cell suspension with a density of 7 × 10^4^ was added to the 96-well plate and incubated in a constant-temperature incubator with 5% CO_2_ at 37 °C. The experiment was started when the cell density was about 80%, and the drug toxicity assay, carambolaside W, was diluted to several different concentrations at 2 μM, 4 μM, 8 μM, 16 μM, 32 μM, 64 μM, 128 μM, and 256 μM and added to the cells, respectively. The CCK8 reagent was added after 48 h, and its absorbance was measured using an enzyme marker [27]. For drug treatment ability, firstly, cells were infected with the virus for 2 h, and then the virus inoculum was removed, and the concentrations of 2 μM, 4 μM, 8 μM, 16 μM, 32 μM, 64 μM, 128 μM, and 256 μM carambolaside W was added to the culture for 48 h, and the absorbance was detected by adding CCK8 reagent Detection of cell viability [28]. 

### 2.6. Detection of Apoptosis

The apoptotic status of MDCK cells was examined by flow cytometry. Firstly, MDCK cells were infected with the H1N1 influenza virus for 2 h. The virus inoculum was removed, and 32 μM of carambolaside W was added for 48 h. The cells were then digested with trypsin and collected in a centrifuge tube [29]. After centrifugation, Annexin V-FITC of 5 μL and PI dye of 10 μL were added, gently mixed, and incubated for 20 min away from light. Then, the apoptosis of cells could be detected by flow cytometry.

### 2.7. Cell Cycle Experiment

MDCK cell suspension at a density of 7 × 10^4^ was first added to the 96-well plate and incubated in an incubator at 37 °C with 5% CO_2_. The experiment started when the cell density was about 80%, and the virus was first added to adsorb for 2 h, after which the virus inoculum was aspirated, and carambolaside W was added for a total of 48 h after incubation [30]. To start the cell cycle experiment, the cells were first digested in six-well plates into centrifuge tubes. After centrifugation, the supernatant was removed, and 1 mL of pre-cooled 70% ethanol was added and left overnight at 4 °C. This step was done to fix the cells. After fixation, 0.5 mL of PI staining solution was added to each tube to fully resuspend the cells for staining, followed by flow cytometry to detect red fluorescence at an excitation wavelength of 488 nm to analyze the effect of carambolaside W on the cell cycle.

### 2.8. Alteration of Mitochondrial Membrane Potential

Firstly, MDCK was incubated in an incubator at 37 °C with 5% CO_2_, and the H1N1 influenza virus was added to infect the cells for 2 h. Then 32 μM of carambolaside W was added to continue the incubation until 48 h, and the cells were washed once with PBS, and then 1 mL of JC-1 staining work was added and incubated for 20 min at 37 °C [31]. After completing the incubation, the cells need to be washed using JC-1 buffer, and this process is repeated twice. Finally, the supernatant was removed, and 2 mL of cell culture solution was added for observation under a fluorescent microscope and further accurate analysis using flow cytometry.

### 2.9. Reactive Oxygen Detection

MDCK cells were cultured in six-well plates, infected with the virus when the cells grew to 80% density, and the drug carambolaside W was added and cultured for 48 h, after which the assay for reactive oxygen species was started, and DCFH-DA was prepared to a final concentration of 10 μM and incubated for 20 min at 37 °C in a cell incubator [32]. After finishing the incubation, the cells were washed three times with pure DMEM to completely remove the DCFH-DA that did not enter the cells. The fluorescent probe DCFH-DA is non-fluorescent itself but can be hydrolyzed to DCFH by intracellular esterases; if ROS are present in the cell, DCFH will be oxidized to DCF and then fluoresce, which can be then observed using fluorescence microscopy. Quantitative experiments were then performed to count the cells in each group and finally to quantify the number of reactive oxygen species in each group according to the number of cells at the end of the enzyme marker assay.

### 2.10. Detection of Western Blot

Western blot can detect the protein content in the cells, and this experiment was used to detect the change in protein amount in MDCK cells infected with H1N1 and after infection with H1N1 by adding carambolaside W [33]. Firstly, the H1N1 influenza virus was added to MDCK cells, and the cells were infected for 2 h. Then 32 μM of carambolaside W was added, and after 48 h, the cells were lysed by adding lysis solution to extract the protein from them. Subsequently, the protein amounts were detected using the BCA method, and the proteins were denatured and stored at −20 °C. After the completion of the preliminary work, electrophoresis can be formally started by adding each group of proteins separately, and the different molecular weight proteins will be separated at a constant voltage of 150 V for 60 min, followed by transferring the membrane at a constant current of 110 V, completing the closure by incubating with the primary antibody at 4 °C overnight and then incubating with the selected specific secondary antibody at 4 °C for two hours the next day, and finally completing the development with the help of the ELC luminescence kit. After getting the target band, take C-PARP as an example. We will crop it to a suitable size, use ImageJ to open the picture, and use the rectangle box to select the band for analysis. There will be four peaks, use the straight-line tool to connect the bottom of the four peaks to separate them into independent peaks, and then use the wand tool to detect each peak that corresponds to a numerical value, which is the grayscale value of the protein. The gray value of the corresponding β-actin of the group was examined using the same method. The gray value of the target protein is compared to the gray value of the corresponding β-actin to arrive at the gray value of each group after homogenization. Finally, each group of proteins was expressed as a ratio of the gray value to the control group to give the final result.

### 2.11. Statistical Analysis

All experiment data was presented as mean ± S.D, for the final display. In addition, SPSS 16.0 statistical software is needed to analyze the data. A *t*-test was used to analyze the difference between the two groups of data. If there is a difference, it is considered to be statistically significant, *p* < 0.05 (*), *p* < 0.01 (**), *p* < 0.001 (***), and *p* < 0.0001 (****).

## 3. Results 

### 3.1. Structural Elucidation of Carambolaside W

**Figure 1:**
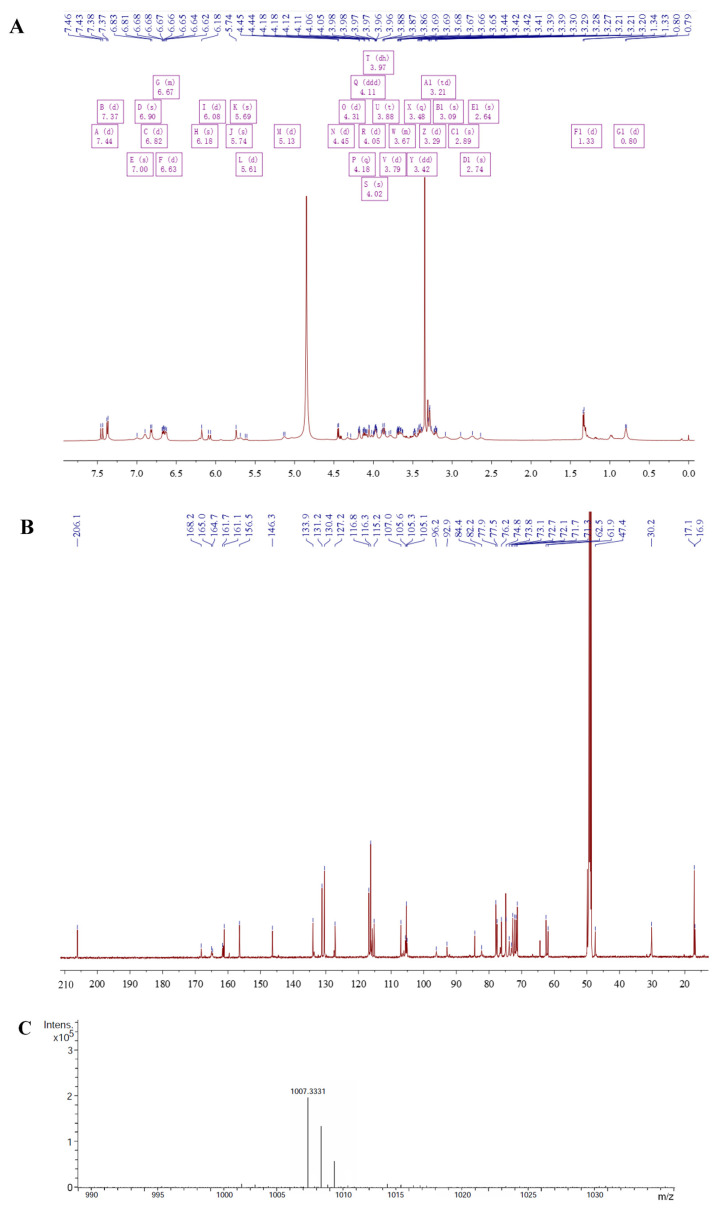
The specific structures of the extracted flavonoid glycosides were obtained by the following experiments, (**A**) This is the H NMR spectrum of the carambolaside W. (**B**) This is the ^13^C NMR spectrum of the carambolaside W. (**C**) This is the HR-EI-MS spectrum of the carambolaside W.

**Figure 2:**
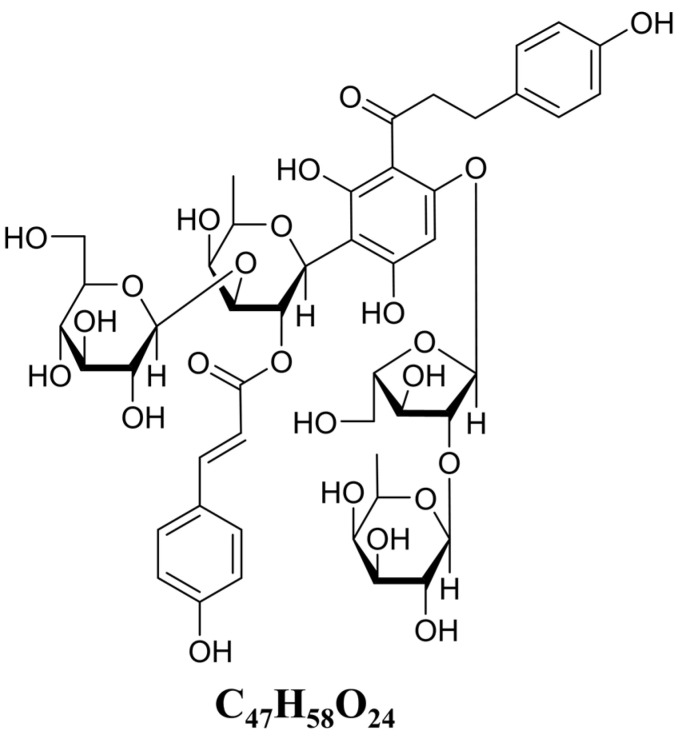
The purchased fresh ripe carambola was subjected to a series of operations such as extraction and elution, and after distillation, the compound was separated by high-performance liquid chromatography and eluted by methanol to obtain the chemical formula and structure as shown above, as deduced from HR-ESI-MS data analysis.

Carambolaside W was deduced to have the molecular formula C_47_H_58_O_24_ from its high-resolution electrospray ionization mass spectrometry (HR-ESI-MS) data in Figure 1, which is 18 Da more than Carambolaside P, a phloretin C-glycoside identified in our previous study [25]. The ^1^H and ^13^C NMR spectra exhibited signals assignable for a phloretin aglycone, four glycosyl moieties, and a *trans*-*p*-coumaroyl moiety (Table 1), which were in good consistency with those of Carambolaside P except for the presence of a glucosyl at the C-3″ instead of a fucosyl in carambolaside W. This deduction could also be confirmed by comparing the NMR data of c-carambolaside W and carambolased N, another phloretin C-glycosides reported in our previous study. Thus, carambolaside W was determined as phloretin 3′-*C*-(2-*O*-*trans*-*p*-coumaroyl-3-*O*-β-d-glucosyl)-β-d-fucosyl-6′-*O*-(2-*O*-β-d-fucosyl)-α-l-arabinofuranoside, and named as carambolaside W, the structural formula is shown in Figure 2. 

### 3.2. Antiviral Ability of Carambolaside W 

**Figure 3:**
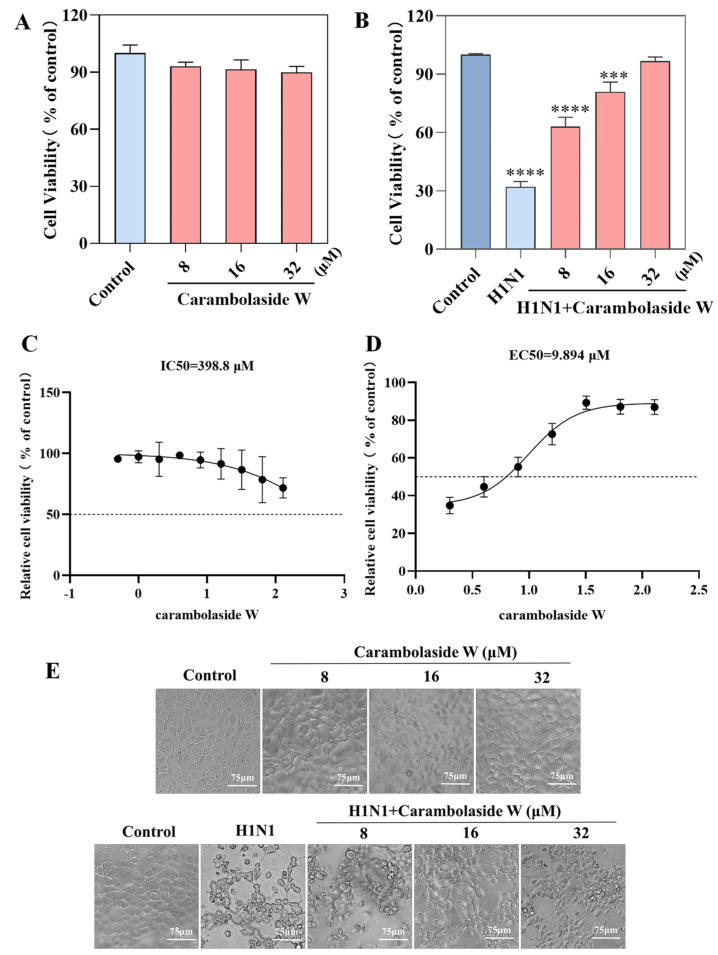
The effect of carambolaside W on the growth of MDCK cells was determined by the CCK8 assay. (**A**) Cytotoxicity assay to detect whether carambolaside W is harmful to MDCK cells. (**B**) Antiviral assay: to detect the inhibitory effect of carambolaside W on infection with H1N1 influenza virus. (**C**) Half maximal inhibitory concentration (IC50) caused by carambolaside W on MDCK cells. (**D**) Half maximal effective concentration (EC50) of carambolaside W on MDCK cells after the addition of H1N1 virus. (**E**) Observation by phase contrast microscopy of the effect of carambolaside W on the morphology of MDCK cells and the morphological effect of carambolaside W on MDCK cells infected with H1N1 influenza virus. Bars with different characters are statistically different at *p* < 0.0001 (****) and *p* < 0.001 (***) vs. the control group. Each experiment was repeated at least three times (n = 3).

The drug toxicity and antiviral ability of carambolaside W were detected by CCK8. In the carambolaside W cytotoxicity assay, different concentrations of carambolaside W were added to MDCK cells at a density of 80%, respectively, then incubated at 33 °C for 48 h. Figure 3A shows that the cell activity of the group with different concentrations of carambolaside W added did not change significantly compared with the control group; carambolaside W has no injurious effect on the cells. The detection of the antiviral ability of carambolaside W, MDCK cells were firstly infected with the H1N1 influenza virus for 2 h, and then the viral inoculum was removed by adding 8 μM, 16 μM, and 32 μM of different concentrations of carambolaside W at 33 °C for 48 h. When CCK8 was added to the assay, the cellular activity of the virus group decreased significantly compared with the Control group, but in the group with different concentrations of carambolaside W added, the cellular activity gradually increased with the increase in drug concentration, and when 32 μM of carambolaside W was added, its cellular activity was close to that of the Control group, as shown in Figure 1B. The IC50 value of carambolaside W was greater than 200 μM, and the EC50 was tested at 9.894 μM, as shown in Figure 3C,D. All the above results proved that carambolaside W had good anti-H1N1 influenza virus ability and was almost non-toxic to MDCK.

### 3.3. Detection of Apoptosis Status after H1N1 Infection

**Figure 4:**
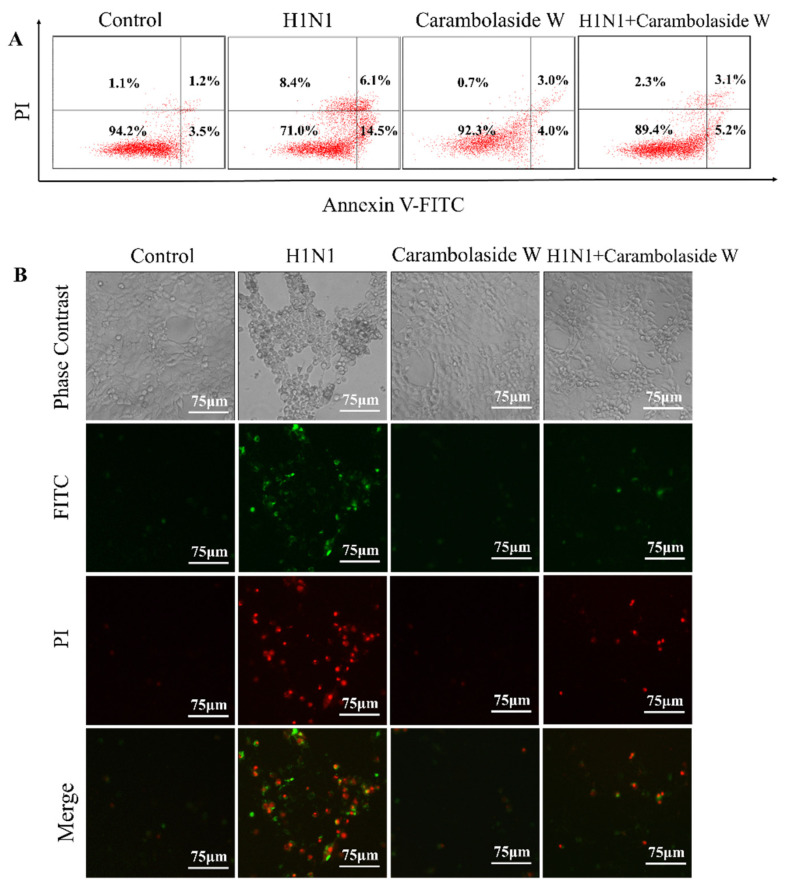
Carambolaside W inhibited H1N1 infection-induced apoptosis regarding Annexin-V/PI staining assay. (**A**) Detection of early and late apoptotic status of cells by flow cytometry. (**B**) Cell morphology and fluorescence intensity were examined using an inverted microscope. Green fluorescence indicates early apoptotic cells and red fluorescence indicates late apoptotic and necrotic cells. The concentration of carambolaside W used here is 32 μM. Each experiment was repeated at least three times (n = 3).

Typical cytopathic effects appeared after MDCK cells were infected with the influenza A H1N1 influenza virus, but this situation changed after carambolaside W was added. The cells were first divided into four groups: control group, virus group, the virus plus drug group, and drug-alone group, by adding Annexin V-FITC and PI fuel to assist in the detection of apoptosis status. MDCK cells were infected with the H1N1 influenza virus, then carambolaside W drug was added and incubated at 33 °C for 48 h. The cells were analyzed by flow cytometry for the regulation status. As shown in Figure 4A, the percentage of early and late apoptotic cells in the Control group was 3.5% and 1.2%, respectively, which might be slightly affected by TPCK trypsin in the virus maintenance solution, and the percentage of early and late apoptotic cells in virus group with H1N1 added was 14.5% and 6.1%, respectively. It can be found that the number of apoptotic cells increased after infection with the virus, and the percentage of cells with early apoptosis and late apoptosis was 4.0% and 3.0%, respectively, when 32 μM carambolaside W was added to MDCK cells only, showing that the cells grew normally. In the group with H1N1 influenza virus and carambolaside W added at the same time, the percentage of cells with early apoptosis and the percentage of early apoptotic and late apoptotic cells were 5.2% and 3.1%, respectively, in the group with the addition of H1N1 virus alone, which showed a decrease in the percentage of early apoptotic and late apoptotic cells, again demonstrating that carambolaside W could indeed play a good role in inhibiting the cell regulation induced by H1N1 infection.

### 3.4. Effects of Carambolaside W on Cell Cycle

**Figure 5:**
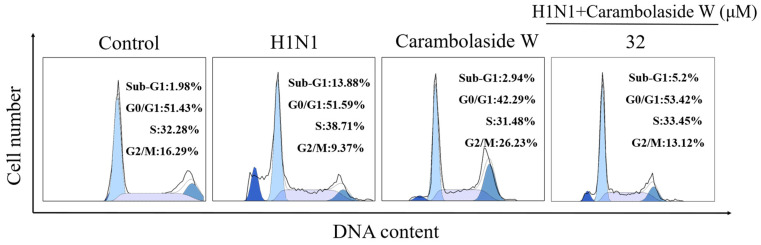
Cell growth cycle distribution after H1N1 influenza virus infection was examined using flow cytometry, as well as the effect of adding carambolaside W on cell life cycle distribution. The concentration of carambolaside W used here is 32 μM.

In order to explore how carambolaside W can inhibit the apoptosis induced by the H1N1 influenza virus, the cell cycle of carambolaside W was tested in this study, which was also divided into four groups: control group, virus group, group with both viruses and the drug, and drug-only group. As shown in Figure 5, the percentage of sub-G1 peak in the viral group is 13.88%, which is much higher than that in the control group. However, after adding carambolaside W, the percentage of sub-G1 peak decreased compared with that in the viral group. Carambolaside W actually effectively inhibits apoptosis induced by H1N1 influenza virus infection. As shown in Figure 5, the proportion of cells in the G2/M phase in the added H1N1 virus group decreased compared with that in the control group [34]. However, after the addition of carambolaside W, the proportion of cells in the G2/M phase increased, and the proportion was close to that in the control group. It is hypothesized that H1N1 virus infection will cause the cells to stay in the synthesis phase of DNA, not enter the final division, and then go into apoptosis, but the addition of carambolaside W can make the cells’ life no longer end in the synthesis phase and even help them complete the division to form daughter cells to survive normally. From the detection of the cell cycle, we can find that carambolaside W inhibits H1N1 influenza virus-induced apoptosis, probably by promoting the completion of cytokinesis.

### 3.5. Effects of H1N1 Infection of MDCK on Mitochondria

**Figure 6:**
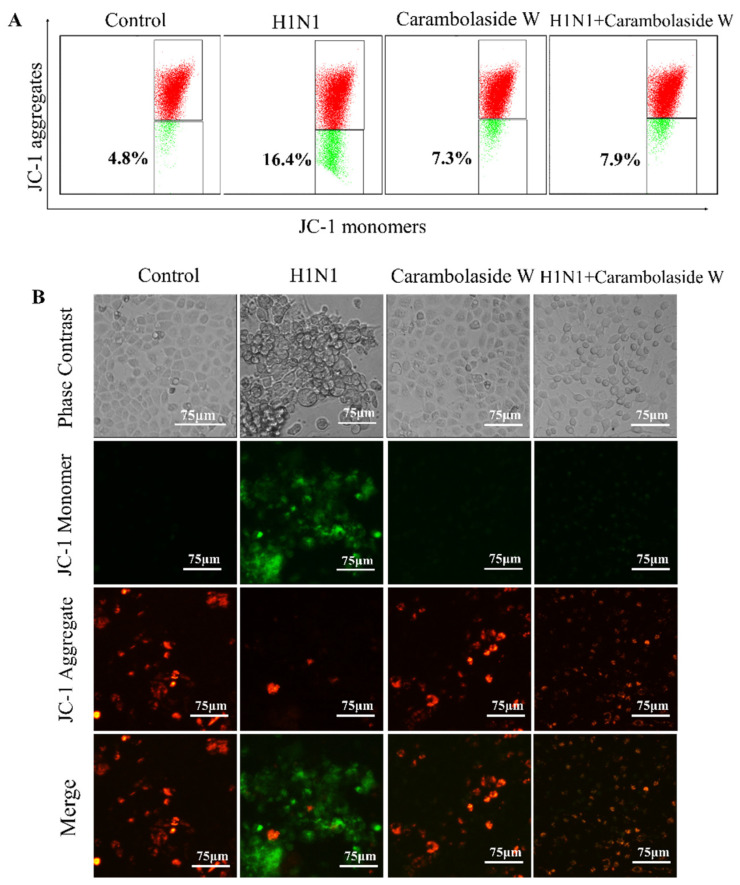
Effect of carambolaside W on the cellular mitochondrial membrane potential of MDCK cells infected with H1N1 influenza virus. (**A**) Comparison of the mitochondrial membrane potential of MDCK cells infected with H1N1 influenza virus and the change of MDCK mitochondrial membrane potential after adding carambolaside W. (**B**) Inverted microscope detection to observe the change in mitochondrial membrane potential. At high mitochondrial membrane potential, J-aggregates are formed and show red fluorescence; at low mitochondrial membrane potential, JC-1 is monomeric and shows green fluorescence. The concentration of carambolaside W used here is 32 μM, each experiment was carried out a minimum of three times (n = 3).

Mitochondria are an important energy center in the cell that can provide the energy required by the cell through the ATP produced by the respiratory chain [35]. The membrane potential of mitochondria is also one of the important indicators for maintaining the normal function of mitochondria, and when the membrane potential of mitochondria is changed, the apoptotic proteins on its inner membrane will be released, causing apoptosis [36]. It can be seen from Figure 6A that MDCK cells were infected with H1N1 after the decrease in mitochondrial membrane potential occurred, and the amount of JC-1 monomer increased from 4.8% to 16.4%. However, after carambolaside W is added, the amount of JC-1 monomer drops to 7.9%, which is consistent with the results in Figure 6B. When H1N1 is added, the green fluorescence is abundant, and when carambolaside W is added, the green fluorescence almost disappears, which inhibits the decline of mitochondrial membrane potential. It was suggested that carambolaside W could inhibit apoptosis induced by the influenza virus by modifying mitochondrial membrane potential.

### 3.6. Changes of Reactive Oxygen Species Produced by Carambolaside W

**Figure 7:**
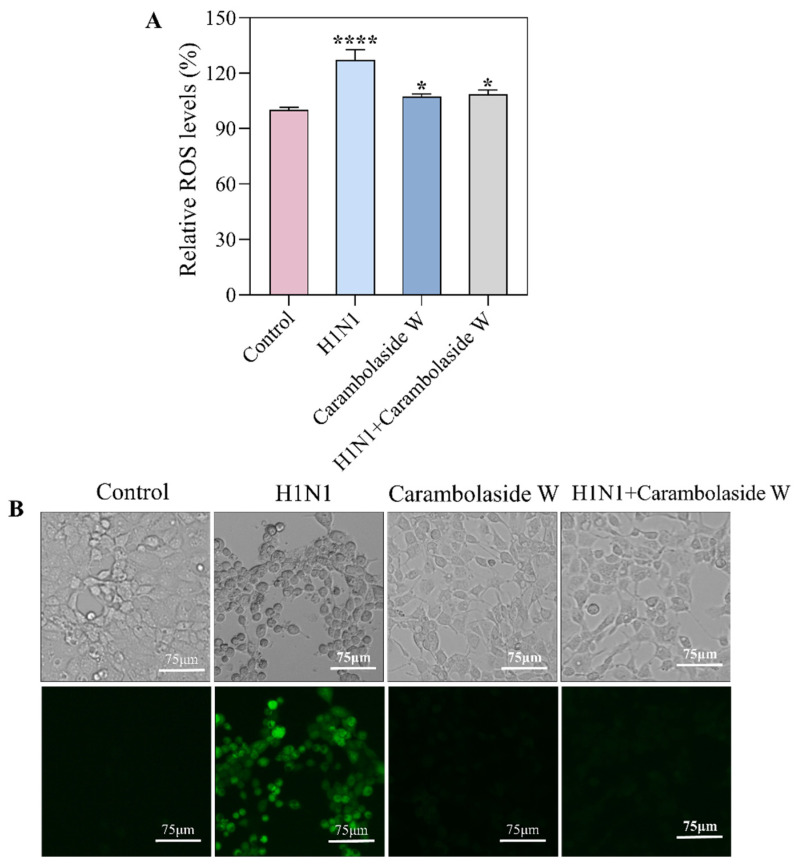
Carambolaside W inhibited ROS production in MDCK cells after infection with the H1N1 influenza virus. (**A**) Enzyme marker was used to assess the intensity of DCF fluorescence generated by intracellular ROS oxidation. (**B**) Inverted microscopy was used to observe MDCK cells and the changes in the intensity of DCF fluorescence expression therein. The concentration of carambolaside W used here is 32 μM. Using the one-way ANOVA de-calculate and Bars with different characters are statistically different at *p* < 0.0001 (****) and *p* < 0.05 (*) vs. control group. Each experiment was repeated at least three times (n = 3).

Mitochondria are important mediators of cellular metabolism, regulators of ATP levels, and calcium homeostasis; they can also be producers and targets of ROS. Previous experiments have found a decrease in mitochondrial membrane potential after infection with the H1N1 influenza virus. The study also corresponds to Figure 7A, which is a quantitative assay of ROS, and it can be seen that ROS production increased significantly after the addition of the H1N1 virus, but cellular function returned to normal after the addition of carambolaside W [37]. In addition, the fluorescent probe DCFH-DA was used, and intracellular ROS can oxidize DCFH to DCF to emit green fluorescence. As can be seen in Figure 7B, after infection with the H1N1 influenza virus, the intracellular green fluorescence increased substantially, indicating that the number of ROS produced in the cell at this time did increase, but then the green fluorescence almost disappeared after the addition of carambolaside W. The above results indicate that carambolaside W can not only reverse the decrease in mitochondrial membrane potential brought about by H1N1 infection but also inhibit the excessive production of intracellular ROS and the regulation of oxidative stress after viral infection, thereby maintaining normal cell survival.

### 3.7. Effect of Carambolaside W on Protein Expression

**Figure 8:**
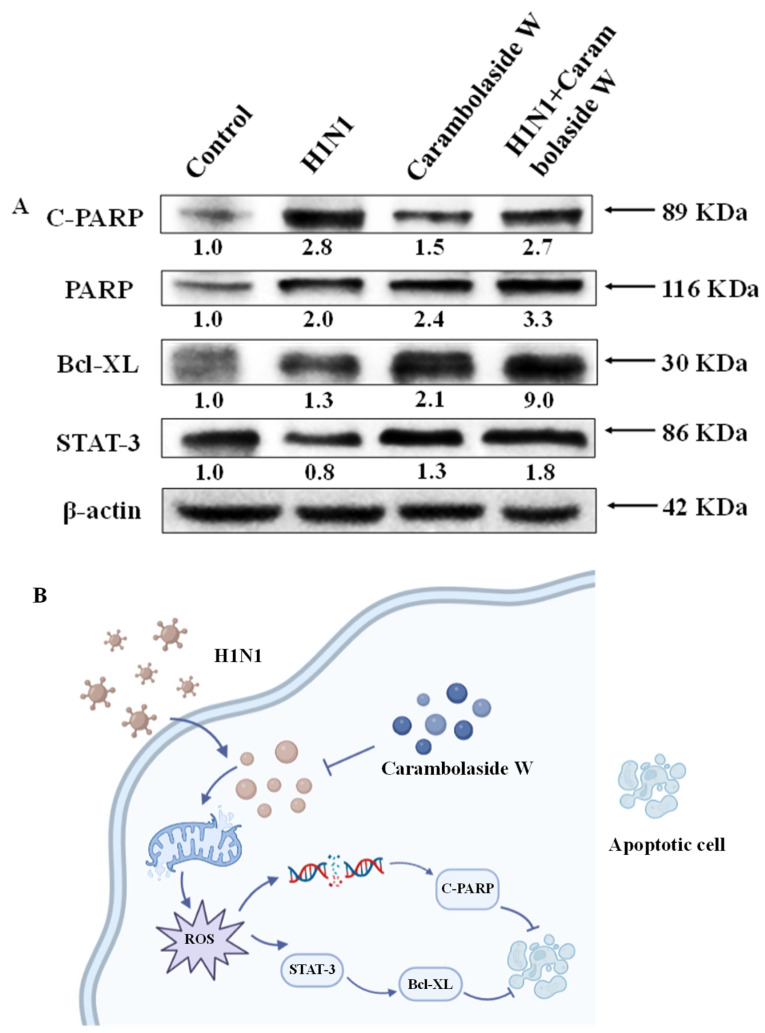
Effects of carambolaside W on intracellular protein signaling pathway expression. (**A**) Western blot detection of PARP, Bcl-XL, and STAT-3 protein expression. (**B**) Carambolaside W was involved in the changes in the apoptotic signaling pathway in H1N1-infected MDCK cells, and its main effects were the mitochondria-related signaling proteins Bcl-XL and PARP and the cytokine-stimulation-related protein STAT-3. The concentration of carambolaside W used here is 32 μM.

Since carambolaside W apparently inhibits H1N1 influenza virus-induced apoptosis, it is speculated whether carambolaside W would modulate proteins in cells. Among the Bcl-2 protein family, Bcl-XL is located outside the mitochondrial membrane and inhibits cytochrome c release [38,39]. Cytochrome c is located in the lumen between the inner and outer mitochondrial membranes. Stimulation of apoptotic signals causes cytochrome C to be released from the mitochondria into the cytoplasm, which then initiates a cascade of reactions in the caspases, causing apoptosis. As shown in Figure 8A, it can be seen that the expression of Bcl-XL was increased in the group with the addition of carambolaside W compared with the H1N1 group. The gray value of Bcl-XL increased from 1.3 to 9.0. PARP is an enzyme closely related to DNA repair that recognizes structurally damaged DNA fragments and activates them. PARP can be sheared by a variety of caspases *in vitro*. After infection with the H1N1 influenza virus, it can be seen that the gray value of C-PARP is 2.8, with a corresponding increase in gray value relative to the control group, but after the addition of carambolaside W, the gray value of C-PARP decreases to 2.7. STAT-3 is also a protein that can be effective against apoptosis. Its protein gray level for H1N1 virus infection was 0.8, but the protein gray level of STAT-3 increased to 1.8 after the addition of carambolaside W. Figure 8B shows the mechanism of carambolaside W against the H1N1 influenza virus. Synthesize the above results to show that carambolaside W can indeed mobilize intracellular mitochondria and apoptosis-related proteins to regulate cell survival.

## 4. Discussion

Influenza is an acute respiratory infection caused by the influenza virus, the incidence of which varies by region and time of year. Because of its potential rapid spread and high incidence, it is highly transmissible in the population and can be disseminated, epidemic, and sometimes pandemic, and it is reported that about 250,000–350,000 people die from influenza each year worldwide [40]. Influenza A virus is the most contagious and pathogenic, often spreading by mouth, nose, air, droplets, etc. Patients may experience cough, sore throat, body aches and weakness, headache, high fever, etc. In mild cases, the disease is often limited to the upper respiratory tract, but the risk of pulmonary infection is also high in people with low immunity, such as the elderly, children, and people with immunodeficiency diseases [41]. 

Vaccination is currently the main measure to prevent infection, while for post-infection treatment of influenza viruses, there are three registered drug types specifically targeting influenza viruses, M2 proton channel antagonists (amantadine), neuraminidase inhibitors (zanamivir, oseltamivir), and, in addition, the first polymerase inhibitor favipiravir (T-705) was recently approved in Japan for the treatment of influenza [42]. However, the long-term use of these drugs has brought about the emergence of drug-resistant strains due to the single targeting of the drugs, and the high susceptibility of influenza virus surface antigens to mutation has made the development of anti-influenza drugs an ongoing concern [43,44].

Influenza virus infection affects normal cellular life processes such as protein synthesis, proliferation, etc. Apoptosis is thought to be an innate cellular response in the face of an invading pathogen, and its apoptosis contributes to the onset of virus replication and propagation, characterized by the occurrence of nuclear fragmentation, cell shrinkage, and caspase activation [12]. In response to apoptosis following influenza virus infection, a search has been made for drugs that directly inhibit this apoptosis and thus abort the continued replication of the virus in the cell as well as its spread.

In this study, we extracted a natural flavonoid glycoside compound from carambola and investigated whether this natural compound could effectively inhibit apoptosis. Firstly, we analyzed the extracted natural glycoside compound by HR-ESI-MS and other analyses, obtained its chemical formula as C_47_H_58_O_24_, and named it carambolaside W. The natural extract was tested and found to exhibit low toxicity in MDCK cells, and it was effective in inhibiting the replication of the influenza virus. Subsequently, the apoptosis was examined by flow cytometry, and it was found that the early apoptosis, as well as the late apoptosis of the cells, increased to some extent after infection with the H1N1 influenza virus, but after the addition of carambolaside W, it was found that the early and late apoptosis conditions of the cells were reduced accordingly [45]. When cells undergo apoptosis, their DNA fragmentation often occurs along with it, and the DNA damage will cause the normal life cycle of cells to come to a standstill. It can be seen that the proportion of cells stagnating in the S-phase increases after H1N1 influenza virus infection, but after the addition of carambolaside W, it is found that the proportion of cells in the S-phase returns to a similar proportion to the control group, which again proves that carambolaside W can indeed inhibit the DNA fragmentation brought about by influenza virus infection and restore the cells to a healthy state.

Mitochondria are important energy centers in cells, and their membrane potential is one of the important indicators of maintaining normal mitochondrial function. When the mitochondrial membrane potential is changed, it will cause the release of apoptotic proteins on the inner membrane surface and affect the state of the cells [46]. Bcl-2 family members play an important role in subsisting the apoptosis in mitochondrial membranes [47]. Bax supports apoptosis, while Bcl-XL binds to Bax, inactivating it and interfering with the process, then inhibiting apoptosis [39]. Western blot showed that the gray value expression of Bcl-XL increased from 1.3 when the virus was added to 9.0 after the addition of carambolaside W, indicating that carambolaside W could effectively increase the expression of Bcl-XL to inhibit apoptosis induced by the H1N1 virus. Meanwhile, the JC-1 experimental assay showed that the mitochondrial membrane potential decreased significantly after virus infection, and a large green fluorescence could be seen under the microscope. However, after the addition of carambolaside W, the green fluorescence was reduced, and the red fluorescence showing high mitochondrial membrane potential was restored. Mitochondria induce apoptosis, and their mediated apoptosis is closely related to the production of intracellular ROS. Excessive production of ROS can disrupt the oxidative homeostasis of cells and lead to loss of cellular integrity and function, as seen in the increased production of intracellular ROS after infection with the H1N1 influenza virus, but carambolaside W effectively inhibited the production of ROS and maintained the homeostasis of the oxidative homeostasis of cells, thus inhibiting apoptosis. Signal transducer and activator of transcription 3 (STAT3) is an important transcription factor, and multiple biological activities in cell proliferation, survival, apoptosis, and differentiation in a wide range of cell types [48]. In previous studies, many therapeutic drugs targeting STAT3 signaling have also been developed, but most of the drugs targeting STAT3 expression usually have problems of high cytotoxicity and poor pharmacokinetics [49]. In contrast, natural products are more favored to be used as drugs that can alter STAT3 activity, which is not only safe but also mostly easy to obtain. Flavonoids such as curcumin and resveratrol are effective in altering STAT3 activity [50]. Carambolaside W, a natural flavonoid glycoside, was also tested in this study to see if it had any effect on STAT3 activity. Western blot results showed that after infection with H1N1 influenza, the viral infection affected the STAT3 activity, and its gray value expression was 0.8, but after the addition of the compound carambolaside W, it exerted an anti-apoptotic effect, and the expression of the STAT3 protein was increased to 1.8. It was again verified that carambolaside W did have the effect of inhibiting H1N1 influenza virus-induced apoptosis and damage, and this inhibition was achieved by modulating the BCL-XL and STAT-3 signaling pathways.

The present study showed that carambolaside W reduced mitochondrial damage and induced apoptosis by inhibiting the overproduction of ROS and regulating the expression of apoptosis-related proteins. In addition, the regulation of STATE-3 protein expression by carambolaside W suggests that it may inhibit clinical symptoms such as pneumonia caused by inflammatory cytokine production after influenza virus infection, which is subject to follow-up studies [51]. In conclusion, carambolaside W, as a natural flavonoid glycoside extract, has low toxicity, can effectively inhibit influenza virus replication, and can inhibit apoptosis, making it a potential drug for the effective treatment of influenza virus.

## 5. Conclusions

In this study, the experimental results lead to the following conclusions: Carambola has been considered a high-quality source of various minerals and natural antioxidants with high nutritional value and antioxidant and anti-inflammatory capacity. Carambolaside W, a novel flavonoid glycoside extracted from carambola, was used in the treatment of the H1N1 influenza virus. Carambolaside W showed good effects in inhibiting influenza virus replication, both in the mitochondrial membrane potential assay and the ROS assay, and it could be found that after the addition of carambolaside W, the mitochondrial membrane potential and ROS expression levels were basically restored to normal levels. The antiviral mechanism also showed that carambolaside W could effectively regulate the expression of STAT-3 and Bcl-XL proteins to inhibit oxidative stress and the onset of apoptosis caused by H1N1 virus infection. Carambolaside W can also regulate C-PARP to repair DNA damage. In conclusion, carambolaside W has shown excellent anti-H1N1 ability and is a potential drug that can effectively treat the H1N1 influenza virus.

## Figures and Tables

**Table 1:** ^1^H (500 MHz) and ^13^C (125 MHz) NMR data of carambolaside W in CD_3_OD.

Carambolaside W					
H/C	*δ*_H_ (*J* in Hz)	*δ*_C_	H/C	*δ*_H_ (*J* in Hz)	*δ*_C_
1		133.9	5‴	3.37, m	77.5
2	6.90/7.00, br s	130.4	6‴	3.87, dd (12.1, 4.0)	62.5
3	6.63/6.67, d (6.9)	116.3		3.68, dd (12.1, 3.5)	
4		156.5	1⁗		127.2
5	6.63/6.67, d (6.9)	116.3	2⁗	7.37, d (8.4)	131.2
6	6.90/7.00, br s	130.4	3⁗	6.82, d (8.4)	116.8
7	2.74/2.64, br s (2H)	30.2	4⁗		161.1
8	3.36/3.09, br s (2H)	47.4	5⁗	6.82, d (8.4)	116.8
9		206.1	6⁗	7.37, d (8.4)	131.2
1′		105.3	7⁗	7.44, d (15.9)	146.3
2′		165.0	8⁗	6.08, d (15.9)	115.2
3′		105.3	9⁗		168.2
4′		164.7	A1	5.74, br s	107.0
5′	6.18 (s)	96.2	A2	4.18, m	92.9
6′		161.7	A3	4.12, dd (7.8, 4.8)	76.2
1″	5.13, d (10.2)	73.8	A4	3.97, m	84.4
2″	5.69, br s	71.7	A5	3.64, m	61.9
3″	3.97, m	82.2		3.79, br d (11.1)	
4″	4.02, br s	73.1	F1	3.96, m	105.1
5″	3.87, m	76.2	F2	3.39, m	72.7
6″	1.33, d (6.3, 3H)	17.2	F3	3.25, m	72.1
1‴	4.45, d (7.7)	105.5	F4	3.42, m	74.8
2‴	3.29, m	76.2	F5	2.89, br s	71.7
3‴	3.31, m	77.9	F6	0.80, br d (6.2)	16.9
4‴	3.35, m	71.3			

*δ*: chemical shift in ppm, *J*: coupling constant, s: singlet, br s: broad singlet, d: doublet, br d: broad doublet, dd: doublet of doublet, m: multiplet.

## Data Availability

Data sharing is not applicable to this article as no datasets were generated or analyzed during the current study.
